# Plant viral suppressors HcPro and P19 inhibit CRISPR-Cas13-mediated transcript interference

**DOI:** 10.1007/s44154-026-00339-9

**Published:** 2026-09-28

**Authors:** Veerendra K. Sharma, Sandeep Marla, David E. Cook

**Affiliations:** 1 Department of Forest Ecosystems and Society, Oregon State University, Corvallis, OR USA; 2 Department of Plant Pathology, Kansas State University, Manhattan, KS USA; 3 Department of Agronomy, Kansas State University, Manhattan, KS USA

**Keywords:** Anti-CRISPR, CRISPR-Cas13, GIGS, Viral suppressors of RNA silencing, RNAi

## Abstract

RNA plays a central role in cellular dynamics, mediating the formation of proteins from genetic information. Thus, there is great interest in technologies that can alter RNA content of a cell to achieve desired outcomes. RNA targeting CRISPR‒Cas13 enables precise, sequence-guided targeting of RNA to disrupt translation and can therefore be used for cellular engineering. However, guide RNA from CRISPR systems can silence target transcripts independent of the Cas13 protein, complicating the deployment of CRISPR-Cas13 for biotechnology. To prevent Cas13-independent silencing and encourage Cas13-mediated transcript reduction, we explored the use of viral suppressors of RNAi. Here, we report that expression of two viral suppressors of RNAi, HcPro and P19, unexpectedly blocked Cas13-mediated transcript silencing in addition to Cas13-independent RNAi-like silencing. To further understand how HcPro blocks Cas13-mediated silencing, we expressed an RNA silencing-deficient HcPro mutant for RNA-targeting assays. The results demonstrate that mutant HcPro failed to suppress Cas13-dependent silencing, indicating that HcPro RNAi suppression also interferes with Cas13 silencing. These results suggest that plant viruses may be primed to overcome engineered CRISPR-Cas13 antiviral resistance through the activity of previously evolved RNAi silencing suppressors.

**Supplementary Information:**

The online version contains supplementary material available at https://doi.org/10.1007/s44154-026-00339-9.

## Introduction

Genome engineering offers a powerful and time-efficient approach in modern biotechnology to drive advancements in crop improvement and precision breeding. Several different genome engineering tools, such as zinc finger nucleases (ZFNs) (Shukla et al. 2009; Townsend et al. 2009; Marton et al. 2010), TAL effector nucleases (Cermak et al. 2011a, b; Li et al. 2012; Zhang et al. 2013; Shan et al. 2013), and CRISPR-Cas9 (Li et al. 2013; Shan et al. 2013; Schiml et al. 2014; Cai et al. 2020), have been utilized for targeted gene editing in multiple plant species. However, gene editing causes permanent alterations at the genomic level and may result in unintended editing at the non-target locus (Guo et al. 2023). In contrast, transcriptome engineering provides a flexible, spatiotemporal regulation of RNA for crop improvement and virus resistance (Canto-Pastor et al. 2019; Zhou et al. 2020; Sharma et al. 2015). Conventional RNA targeting tools in plants include antisense RNA (Kawchuk 1991; Smith et al. 2000), hairpin RNA (Wesley et al. 2003; Helliwell and Waterhouse 2003), and artificial microRNAs (amiRNA) (Schwab et al. 2006). These technologies exploit endogenous RNA interference (RNAi) pathways, but can produce off-target silencing (Jackson et al. 2003), which can limit their application. Additionally, antiviral strategies based on antisense RNA, hairpin RNAi, and miRNA technologies can be overcome by viral proteins that act as viral suppressors of RNAi (VSRs) (Anandalakshmi et al. 1998; Li and Ding 2001). Identified type VI CRISPR-Cas13 systems (East-Seletsky et al. 2016; Konermann et al. 2018) have emerged as an alternative tools for specific RNA cleavage. (Abudayyeh et al. 2016, 2017; Sharma et al. 2022), base editing of target transcripts (Abudayyeh et al. 2019), pathogen detection in animals (Gootenberg et al. 2017; Shen et al. 2020), and insect cell lines (Tng et al. 2020), and as antiviral tools in plants (Aman et al. 2018; Zhang et al. 2019; Mahas et al. 2019; Sharma et al. 2021, 2022; Zhan et al. 2023).

We previously demonstrated that both transient and stable expression of 28 nucleotide (nt) long CRISPR RNA (i.e. guide) could cause significant transcript silencing in the absence of the Cas13 protein (Sharma et al. 2022). We termed this phenomenon as guide-induced gene silencing (GIGS) and demonstrated that it likely functions using components of the RNAi machinery. Similar RNAi- like Cas13-independent transcript silencing by CRISPR guides were also observed in human cell line (Blanchard et al. 2021) and mosquito cells (Tng et al. 2020). These reports suggest that Cas13-independent transcript silencing is not limited to plants, but may be a widespread phenomenon in eukaryotes. The occurrence of Cas13-independent RNAi like target silencing could result in off-target effects and undermine the applications of CRISPR-Cas13. Thus, further understanding the Cas13-independent silencing phenomena and developing strategies to mitigate it are important for the deployment of Cas13 for trait engineering.

VSRs are specialized viral proteins evolved to neutralize host plant RNAi, the main plant defense system against viral infection (Anandalakshmi et al. 1998; Li and Ding 2001). For this, we tested if VSRs could be used to prevent Cas13-independent silencing due to their ability to suppress RNAi. We hypothesized that expression of a VSR in combination with Cas13 and guide would eliminate Cas13-independent silencing by suppressing RNAi without impacting Cas13 activity. Two well characterized VSRs, helper component protease (HcPro) from TuMV and P19 from Tomato bushy stunt virus (TBSV) were tested for this purpose (Silhavy et al. 2002; Lakatos et al. 2004; Del Toro et al. 2017). Contrary to our initial hypothesis, our results demonstrate that HcPro and P19 inhibited both Cas13-independent and Cas13-mediated transcript silencing. Using an RNA-silencing-defective mutant (AS9) (Garcia-Ruiz et al. 2015), we further demonstrate that HcPro’s anti-CRISPR-Cas13 function is linked to its RNA silencing suppression activity. This report of viral encoded VSRs acting as anti-CRISPR factors demonstrate a further activity of viral VSRs to promote infection.

## Results

### Expression of HcPro inhibits both Cas13-independent and Cas13-mediated TuMV silencing

**Fig. 1:**
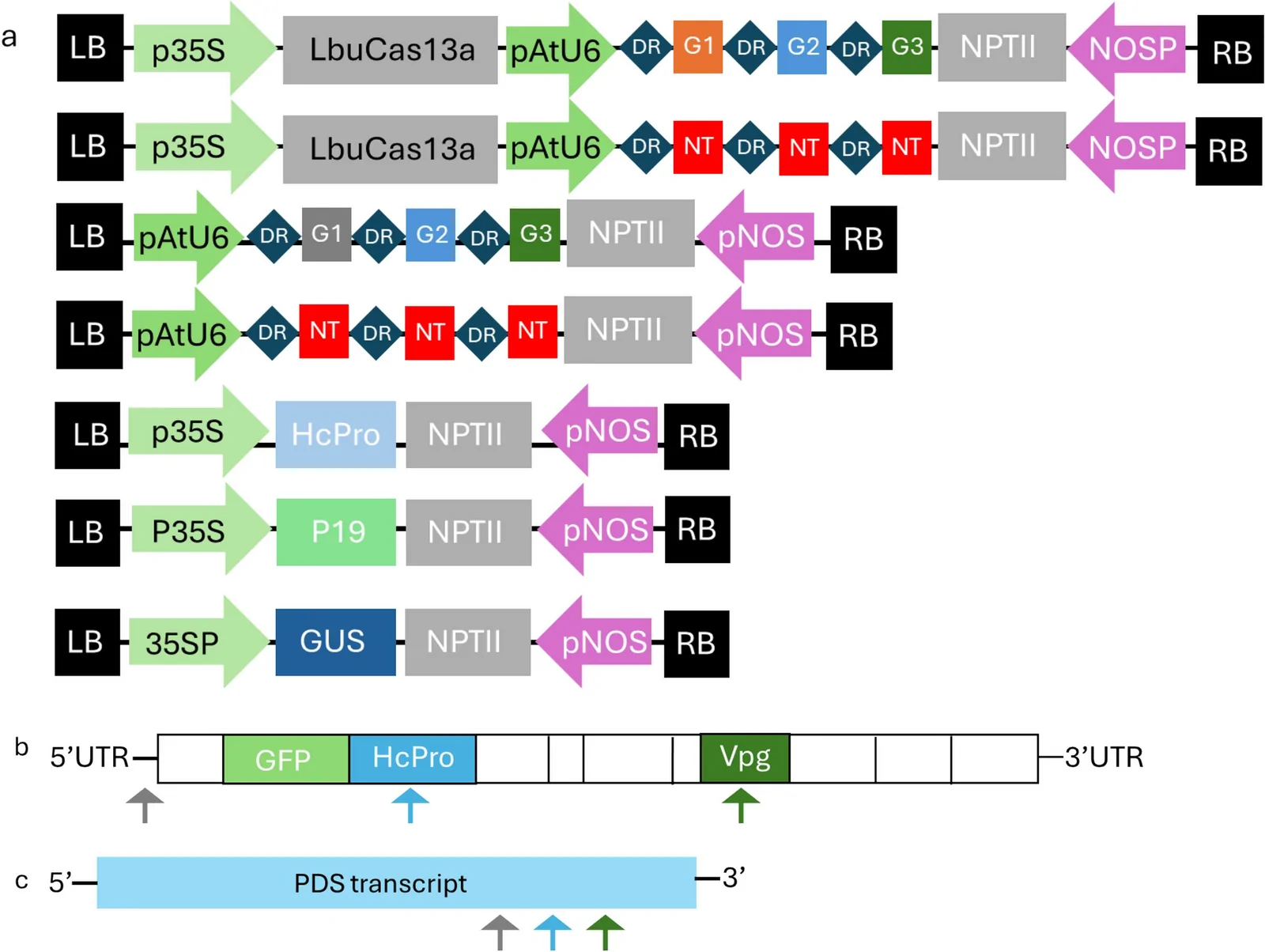
Schematic of T-DNA regions used for transient expression and their RNA targets. **a**, The 35S promoter was used to express LbuCas13, HcPro, P19, and GUS. *Arabidopsis thaliana* U6 promoter (AtU6) was used to express guides, depicted as colored boxes and diamonds. The diamonds represent direct repeat (DR) sequence of Cas13, while colored boxes represent target sequences for the respective target RNA or a non-target (NT) sequence used as a control. TuMV and PDS targeting multiguide construct with and without LbuCas13 were used for Cas13-mediated and Cas13-independent assay respectively, while the non-targeting multiguide constructs with and without LbuCas13 were used as a control. **b**, A diagram showing TuMV genome engineered to express GFP (TuMV-GFP) and indicating the locations of the three targeting sites of TuMV-targeting multiguide (**c**), A diagram showing PDS transcript indicating the locations of the three targeting sites of PDS targeting multiguide

**Fig. 2:**
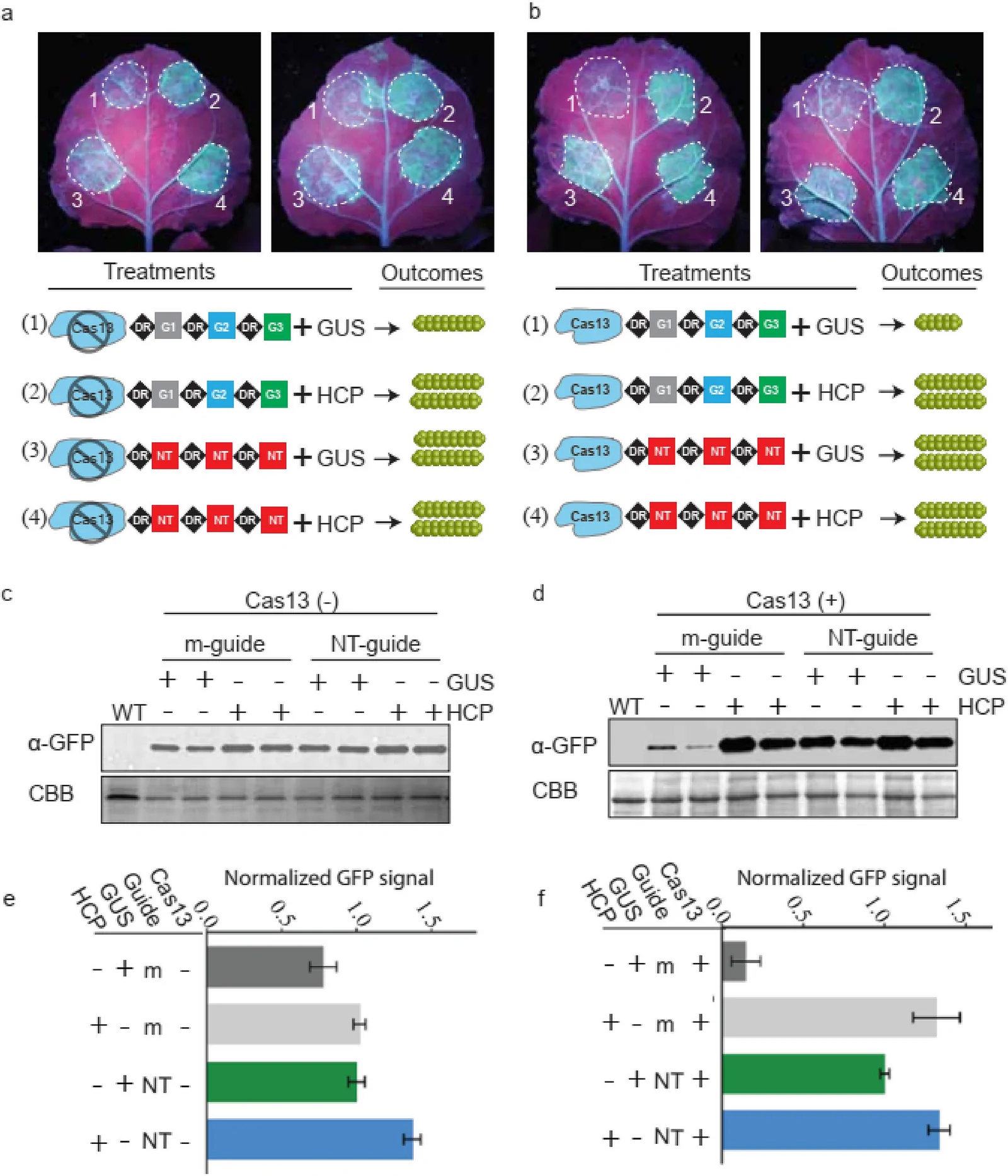
Expression of HcPro inhibits Cas13-independent and Cas13-mediated TuMV interference (**a**, **b**), The accumulation of GFP in Cas13-independent (**a**) and Cas13-mediated (**b**) TuMV interference assays was assessed at 120 h post infiltration using a hand-held UV lamp and the images were taken using a Nikon camera. Agroinfiltrated areas are shown as dashed white circles and numbers represent different treatments shown below the images. **c**, **d**, GFP accumulation in Cas13-independent (**c**) and Cas13-mediated (**d**) TuMV interference assay using antiGFP antibody. For each treatment, two samples were analyzed using western blotting. The Coomassie brilliant blue (CBB) stained gel show roughly equal loading. **e**, **f**, GFP fluorescence in each treatment were quantified using ImageJ software and normalized to the samples expressing GUS and NT and the normalized GFP fluorescence intensity was plotted using R

We aimed to address Cas13-independent transcript silencing using HcPro. To test our hypothesis, we transiently expressed HcPro in Cas13-independent and Cas13-mediated TuMV targeting assays. We expressed a guide with multiple targeting sequences to TuMV, termed a multiguide (m-guide) and a non-targeting multiguide (NT-guide) (Fig. 1a, b). The m-guide or NT-guide were expressed in the presence or absence of Cas13, along with either HcPro or GUS control (Fig. 1a) in *Nicotiana benthamiana* leaves using agroinfiltration (Fig. 2a, b). As expected, in the absence of HcPro (GUS control) m-guide effectively reduced TuMV accumulation as evidence by reduced GFP fluorescence in Cas13-independent and Cas13-mediated assays (Fig. 2a, b spot #1). Expressing GUS and NT-guide did not hamper TuMV accumulation (Fig. 2a, b spot #3). Notably, the expression of HcPro with m-guide inhibited Cas13-independent TuMV-targeting (Fig. 2a spot #1 versus #2). This finding is consistent with our previous study suggesting that Cas13-independent TuMV interference function through RNAi like pathways which are effectively suppressed by HcPro (Sharma et al. 2022). Surprisingly, introduction of HcPro also reduced Cas13-mediated TuMV interference and GFP fluorescence was comparable to the GUS expressing control (Fig. 2b spot #1 versus #2). To confirm the phenotypic observations, we performed western blotting using anti-GFP antibody. Western blot data validated that HcPro expression resulted in substantially higher GFP accumulation compared to expression with GUS (Fig. 2c, d). Densitometric quantification of western blot data using ImageJ revealed that HcPro restored GFP signal by approximately 20% in Cas13-independent assays, and a nearly sixfold increase in GFP accumulation in Cas13-mediated targeting (Fig. 2e, f). To ensure the integrity of our assays, RT-PCR confirmed the absence of Cas13 transcripts in Cas13-negative samples, while showing consistent Cas13 expression in Cas13-mediated assays (Fig. S1) ruling out contamination. Collectively, these data demonstrate that HcPro is a potent inhibitor of both Cas13-independent RNAi-like silencing and Cas13-mediated interference.

## TBSV P19 also inhibits Cas13-independent and Cas13-dependent TuMV interference

**Fig. 3:**
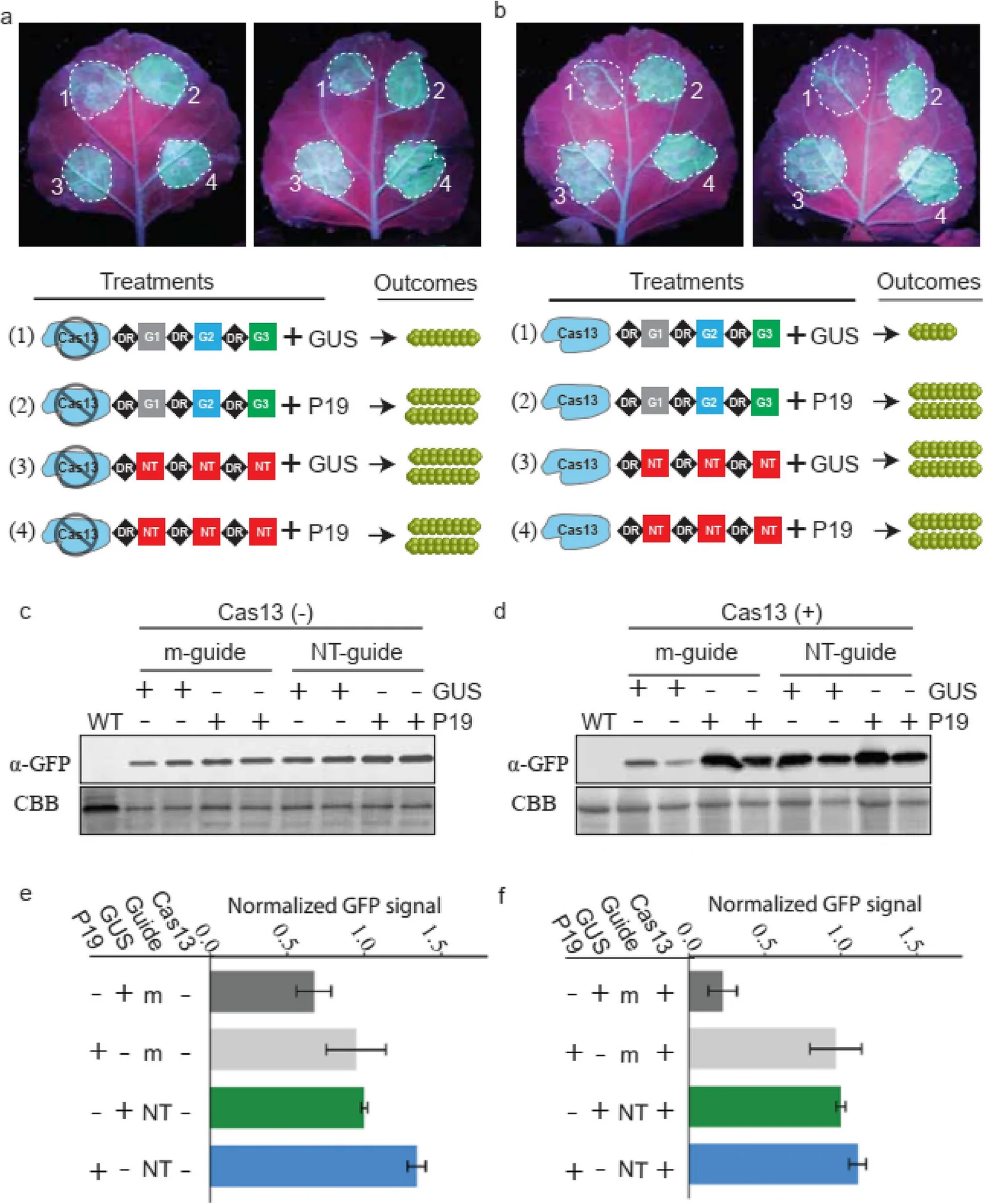
Expression of P19 inhibits Cas13-independent and Cas13-mediated TuMV interference (**a**, **b**), The accumulation of GFP in Cas13-independent (**a**) and Cas13-mediated (**b**) TuMV interference assays was assessed at 120 h post infiltration using a hand-held UV lamp and the images were taken using a Nikon camera. Agroinfiltrated areas are shown as dashed white circles and numbers correspond to different treatments shown below the images. **c**, **d**, GFP accumulation in Cas13-independent (**c**) and Cas13-mediated (**d**) TuMV interference assay respectively using an antiGFP antibody. For each treatment, two samples were analyzed using western blotting. The Coomassie brilliant blue (CBB) stained gel show roughly equal loading. **e**, **f**, GFP fluorescence in each treatment was quantified using ImageJ software and normalized to the samples expressing GUS and NT. The normalized GFP fluorescence was plotted using R

To further characterize if viral silencing suppressors impact Cas13-interference, we expressed P19, a well-characterized VSR from *Tomato bush stunt virus* (TBSV), in both Cas13-independent and Cas13-dependent TuMV-targeting assays using agroinfiltration in *N. benthamiana*. Consistent with our previous experiment, expressing GUS and an m-guide targeting TuMV resulted in very low accumulation of GFP fluorescence, regardless of Cas13 expression (Fig. 3a, b spot #1). However, the introduction of P19 with the same m-guide inhibited Cas13-independent TuMV reduction (Fig. 3a spot #1 versus #2). This finding further confirms that Cas13-independent TuMV interference rely on RNAi-like pathways. Similarly, P19 expression with the m-guide and Cas13 also reduced Cas13-mediated targeting, and resulted in a substantially stronger GFP signal compared to GUS expression (Fig. 3b spot #1 versus #2) In contrast, GUS or P19 with NT-guide did not impact TuMV accumulation (Fig. 3a, b spot #3 and #4). Western blotting to detect GFP accumulation demonstrated that expressing P19 resulted in significantly increased GFP accumulation compared to expressing GUS (Fig. 3c, d). Subsequent densitometric quantification of the GFP bands showed that the expression of P19 caused a 33% and 78% increase in GFP accumulation in the Cas13-independent and Cas13-mediated TuMV targeting assays, respectively (Fig. 3f, g). These results demonstrate that P19 effectively inhibits both Cas13-independent and Cas13-mediated TuMV interference.

## HcPro and P19 inhibit Cas13-independent and Cas13-mediated endogenous transcript silencing

**Fig. 4:**
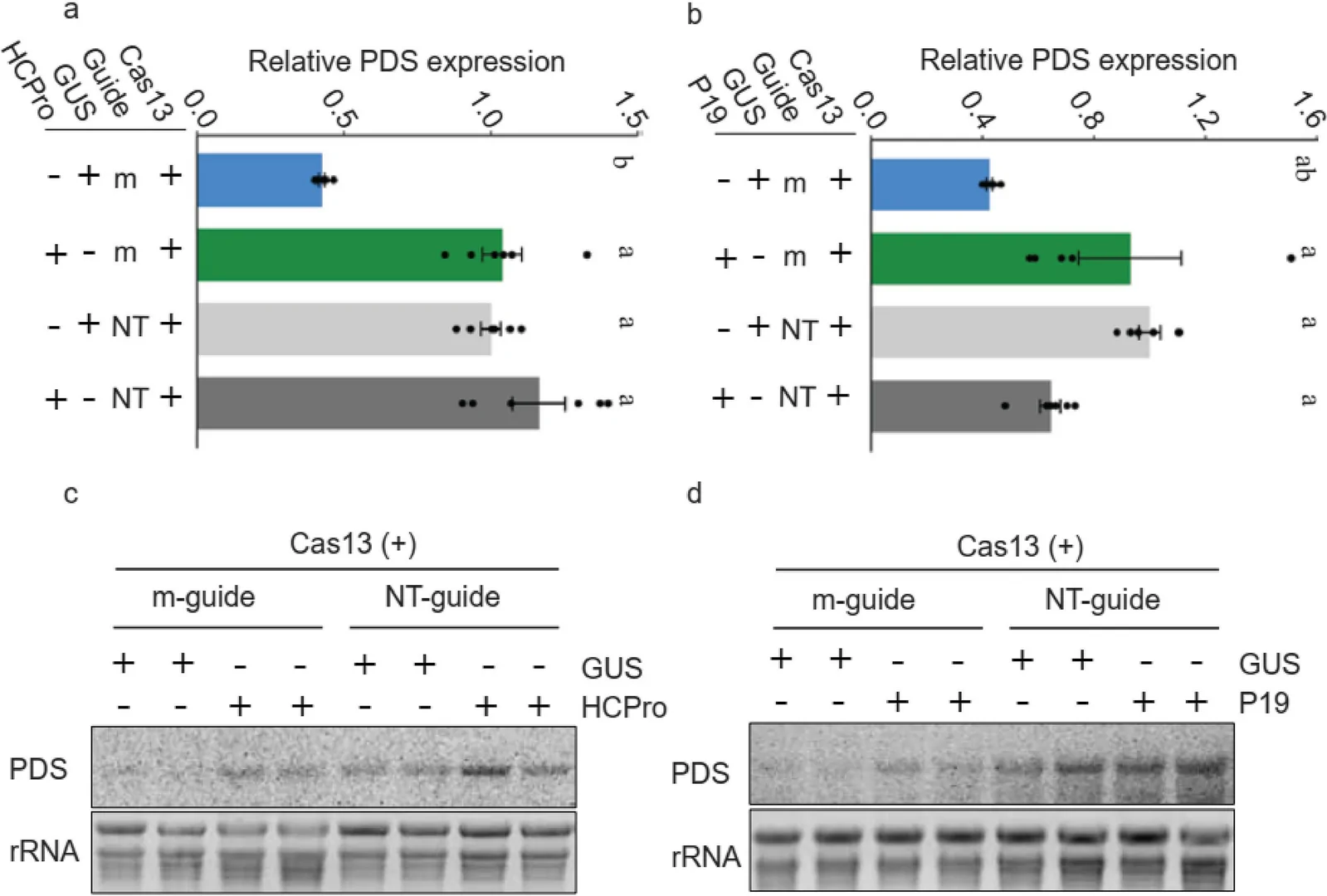
HcPro and P19 inhibit Cas13-mediated endogenous transcript interference (**a**, **b**), Quantification of the PDS transcript using qRT‒PCR. Six independent leaves were used for PDS quantification, and each data point is shown as a black dot with the standard deviation on a bar plot. The samples were standardized to the EF1α endogenous transcript and normalized to the samples coexpressing GUS and NT-guide. **c**, **d**, Northern blots showing PDS transcript levels in the presence of HcPro and P19 respectively. A DIG-labeled PDS-specific probe was used to detect the PDS transcript (PDS panel). SafeStain-stained rRNA was used as a loading control to ensure roughly equal loading. The lanes above the images are labeled to show the presence or absence of different constructs for different treatments. Statistical comparisons were made using one-way ANOVA in R, multiple comparisons of treatments by means of Tukey were performed using the HSD.test function from the Agricolae R package with a *p*-value cut-off of 0.01 for calculating significant difference. Treatments with the same letter are not significantly different

Viruses are known to manipulate host physiology, hijacking cellular machinery for replication, transcription, and systemic movement (Anandalakshmi et al. 1998; Barajas and Nagy 2010; Csorba et al. 2010). Nearly all characterized plant viruses encode VSRs, and it is possible that the anti-Cas13 effects we observed for HcPro and P19 are specifically tied to the context of viral infection. To determine whether HcPro and P19 exert a broader inhibitory effect of Cas13 functionality beyond viral RNA, we tested their impact to reduce transcript reduction of the endogenous target phytoene desaturase (PDS). For these assays, we expressed HcPro and P19 with a PDS targeting m-guide (Fig. 1c) in Cas13-independent and Cas13-mediated assays. We again used a NT-guide and GUS as controls. To quantify the level of PDS transcript in different treatments, we isolated RNA and performed real-time PCR quantification (qPCR). The qPCR data revealed a 60% reduction in PDS transcript when GUS was expressed with m-guide and Cas13 compared to when GUS was expressed with NT-guide and Cas13 (Fig. 4a). In contrast, expressing HcPro or P19 with m-guide and Cas13 showed PDS transcript levels similar to that of the NT-guide and Cas13, demonstrating that HcPro and P19 inhibited Cas13-mediated PDS interference (Fig. 4a, b). Similar observations were seen for Cas13-independent silencing of PDS, where expression of HcPro or P19 compared to GUS resulted in substantially less Cas13-independent interference (Fig. S2). To further confirm these results, we performed northern blotting using a PDS-specific probe to detect PDS transcripts. The northern blot data validated the qPCR results and showed stabilization of PDS transcript in the presence of HcPro or P19 compared to expression with GUS (Fig. 4c, d). Taken together, these results suggest that the inhibitory effects of VSRs on Cas13-independent and Cas13-mediated interference are not limited to viruses and extend to endogenous mRNA targeting. By demonstrating that HcPro and P19 can effectively abolish the knockdown of PDS mRNA by Cas13 protein, we provide evidence that they act as antagonists to Cas13 interference.

## The RNA silencing suppressor activity of HcPro is required for the inhibition of Cas13-mediated transcript interference

**Fig. 5:**
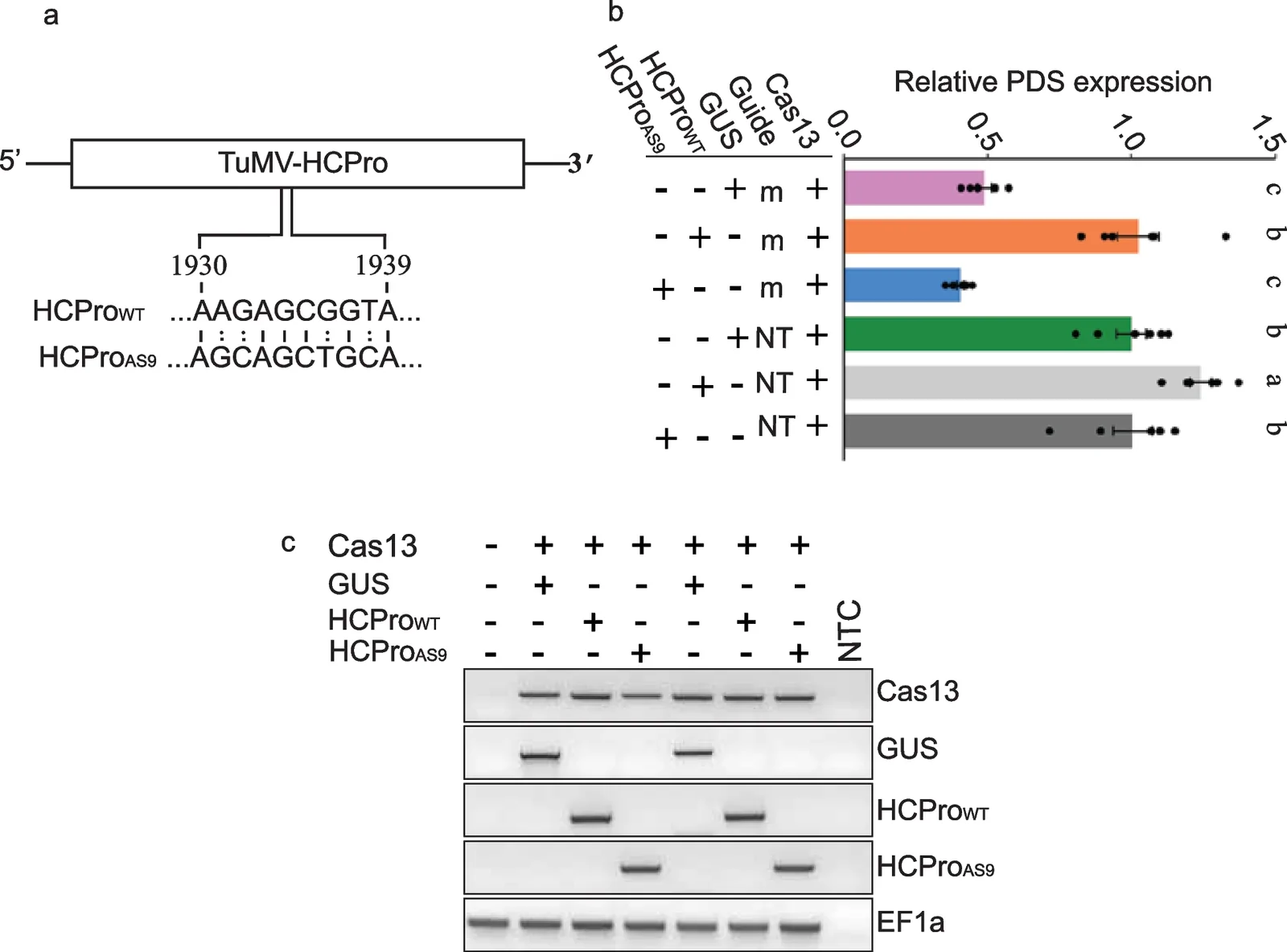
The RNA silencing suppressor activity of HcPro is required for its anti-Cas13 function. **a**, Schematic diagram of the HcPro_AS9_ mutant showing the site of mutagenesis. **b**, Real-time quantification of the PDS transcript. Five independent leaves were used for PDS quantification, and each data point is shown as a black dot with the standard deviation on a bar plot. The samples were standardized to the EF1α endogenous transcript and normalized to the samples coexpressing GUS and NT-guide. **c**, Reverse-transcriptase PCR showing the expression of Cas13, GUS, HcPro_WT_ and HcPro_AS9_ in the respective samples. The EF1α transcript was used as an endogenous control. NTC represents no template control lane (NTC). Statistical comparisons were made using one-way ANOVA in R, multiple comparisons of treatments by means of Tukey were performed using the HSD.test function from the Agricolae R package with a *p*-value cut-off of 0.01 for calculating significant difference. Treatments with the same letter are not significantly different

A fundamentally common feature between RNAi and CRISPR-Cas13 systems is their reliance on short RNA, namely siRNAs and guide RNAs, to achieve target specificity. The potyvirus silencing suppressor HcPro acts by binding and sequestering siRNA (Lakatos et al. 2004; Del Toro et al. 2017), thereby preventing the formation of active RISC complexes. Binding of siRNA by TuMV HcPro requires amino acids in the central domain (Urcuqui-Inchima et al. 2000; Del Toro et al. 2017), and mutations in these amino acids (AS9 mutations) (Garcia-Ruiz et al. 2015) abolish the siRNA-binding activity of HcPro. To investigate whether the anti-Cas13 activity of HcPro is driven by its RNA binding ability, we employed site-directed mutagenesis of HcPro to generate AS9 mutant (HcPro_AS9_) (Garcia-Ruiz et al. 2015). We expressed HcPro_AS9_ or wild-type HcPro (HcPro_WT_) with PDS targeting m-guide in Cas13-mediated PDS interference assay (Fig. 5a). Expressing HcPro_WT_ inhibited Cas13-mediated PDS reduction, resulting in PDS transcript levels statistically similar to that observed for the NT-guide control (Fig. 5b,c). In contrast, expressing HcPro_AS9_ mutant failed to inhibit Cas13-mediated PDS transcript silencing, and PDS transcript levels were significantly lower compared to expressing HcPro_WT_ (Fig. 5b,c). This indicates that HcPro_AS9_ could no longer suppress Cas13-mediated PDS interference. Together, these data indicate that the anti-CRISPR-Cas13 activity of HcPro is dependent on key amino acids shown to be required for RNAi suppression.

## Discussion

Plants use RNAi as the primary defense against viral infection. As a counter defense to RNAi, viruses evolved VSRs to facilitate infection (Li and Ding 2001). VSRs operate through diverse mechanisms, including the sequestration of siRNAs to prevent active RISC assembly or directly interacting with core components of RNAi such as DCLs, AGOs, RDRs, and SGS3 (Li and Ding 2001; Basu et al. 2014). The initial objective of this work was to use VSRs to specifically eliminate Cas13-independent silencing while maintaining Cas13-mediated activity. However, our results revealed that HcPro and P19 unexpectedly inhibited both Cas13-independent and Cas13-mediated silencing in *N. benthamiana*.

Anti-CRISPR (Acr) proteins of phages evolved to overcome the bacterial CRISPR-Cas9 system (Bondy-Denomy et al. 2013) and function through various mechanisms such as causing dimerization of the Cas9 protein, blocking access to the PAM recognition site (Harrington et al. 2017), and interfering with Cas9-crRNA complex (Zhu et al. 2019). While most known Acrs target DNA cleaving CRISPR-Cas9, recent studies have identified Acrs that target CRISPR-Cas13 function by binding to the crRNA-bound effector complex (Meeske et al. 2020) and mimicking the structure of crRNA (Hayes et al. 2025). It is unlikely plant viruses have encountered selective pressure from bacterial CRISPR-Cas systems, but our results show they do have Cas13 inhibitory effects. The results using the HcPro_AS9_ mutant suggest that HcPro interferes with Cas13 guides and could be an indirect consequence of similarities between sRNA of RNAi and Cas13 as both RNAi and Cas13 rely on protein-RNA complex formation. Both HcPro and P19 suppress RNAi by binding double-stranded siRNA intermediates and inhibit the formation of active RISC complexes (delToro et al. 2017; Silhavy et al. 2002), suggesting that VSRs interfere with guide RNA.

Interestingly, previous reports of successful Cas13-mediated viral resistance in plants often targeted the VSR sequences themselves (Aman et al. 2018; Mahas et al. 2019; Gao et al. 2024). Targeting this critical component of the viral genome may have skewed the interaction toward plant immunity by limiting VSR protein accumulation. Our previous study also identified that guide RNA designed against HcPro provided stronger TuMV interference in transient assays compared to guide RNAs against 5’UTR and VPg (Sharma et al. 2022). Similar results reported that targeting viral ORFs other than VSRs provided weaker virus resistance (Aman et al. 2018; Mahas et al. 2019). This suggests that Cas13-mediated antiviral activity in plants is interconnected with native RNAi defense against plant viruses. Contradictor to our results, a previous report showed that CRISPR-Cas13 targeting potato virus Y (PVY) coat protein (CP) provided effective resistance during infection by PVY. This suggests that a VSR could not interfere with Cas13 during *in planta* infection when targeted by transgenic plants expressing CRISPR-Cas13 (Zhan et al. 2019, 2023). However, the same transgenic line (St-CP#7) targeting PVY could not provide PVY-resistance during mixed infection of PVY/PVX (potato virus X), PVY/PVS (potato virus S), or triple infection PVY/PVX/PVS (Zhan et al. 2023). An explanation for why mixed infection broke PVY resistance, and for our results showing VSR co-expression with Cas13 broke resistance, could be that the timing of expression and amount of the Cas13-crRNA components prior to viral infection is critical for determining the interaction outcome. In stably transformed plants, Cas13 and crRNA are present prior to viral infection, potentially allowing the Cas13-crRNA complex to form and target the virus without the accumulation or inhibition by a VSR (Zhan et al. 2019). In our infiltration assays however, Cas13, crRNA, and VSRs are delivered together, allowing their simultaneous expression before TuMV viral inoculation. This simultaneous delivery may allow the VSR to accumulate and inhibit Cas13-crRNA complex formation, resulting in the inhibition of Cas13-mediated virus resistance. This scenario could explain why PVY resistance in line (St-CP#7) was broken by mixed virus infection, suggesting that in the presence of heterologous suppressors from PVS and PVX, the resistance to PVY was compromised (Zhan et al. 2023). Therefore, our results using transient expression shows the potential for VSRs to inhibit Cas13-mediated TuMV resistance when delivered simultaneously, and scenarios where Cas13-crRNA complex formation is inhibited could lead to a loss of resistance. The reduction of Cas13-crRNA in a stable transgenic plant could reasonably occur due to biotic or abiotic stress, or tissue or developmental expression differences. An important takeaway is that while VSRs did not evolve to counter Cas13, mounting results suggest they can exert anti-Cas13 function. This suggests that CRISPR-Cas13-mediated antiviral immunity in crops could break down through multiple scenarios that limit Cas13-crRNA accumulation and through selection of VSR variants with enhanced Cas13-inhibiting function.

## Conclusions

Our results demonstrate that HcPro and P19 function as anti-CRISPR-Cas13 proteins, inhibiting the Cas13-mediated silencing of both a viral target (TuMV) and an endogenous target (PDS). Furthermore, our data indicate that the RNAi-silencing suppressor activity of HcPro is essential for its anti-CRISPR-Cas13 function, suggesting that HcPro interferes with Cas13 by inhibiting CRISPR guide RNAs.

## Materials and methods

### Cloning of constructs

The CRISPR-Cas13 constructs used in this study have been described in detail in Sharma et al. 2022. Briefly, TuMV and PDS targeting m-guides were ordered as oligos and annealed using a thermocycler in oligo annealing buffer (10 mM Tris–Cl pH 8.0, 50 mM NaCl, 1 mM EDTA) (Supplementary Table S1). The annealed oligos were cloned under the *Arabidopsis* U6 promoter using Goldengate cloning. The LbuCas13 sequence was synthesized as gene blocks (IDT, Coralville, IA) and cloned under the 35S promoter in the gateway vector pGWB413 using NEBuilder HiFi DNA Assembly Master Mix (New England Biolabs, Ipswich, MA). For expression of the TuMV HcPro, the HcPro sequence was PCR amplified from TuMV-GFP infectious clone using primers harboring the *Mlu*I and *Avr*II restriction sites and cloned under the 35S promoter in the gateway vector pGWB413. For the expression of tomato bushy stunt virus (TBSV) P19, the sequence was PCR amplified using the TBSV clone as a template with primers harboring the *Mlu*I and *Avr*II restriction sites and cloned under the 35S promoter in the gateway vector pGWB413. For cloning of the GUS gene, the sequence was PCR amplified with primers harboring the *Aat*II and *Avr*II restriction sites and subsequently inserted into the gateway vector pGWB413. For creation of HcPro AS9 mutant, site-directed mutagenesis of HcPro was carried out using a Q5 site-directed mutagenesis kit (New England Biolabs, Ipswich, MA) following the manufacturer’s recommended protocol. Briefly, the HcPro sequence was first amplified and inserted into the cloning vector pJET1.2 (Thermo Scientific, Waltham MA) and subsequently used as a template for site-directed mutagenesis. The mutations were confirmed by sequencing, and subsequently, the mutated HcPro was digested with *Mlu*I and *Avr*II and cloned into the gateway vector pGWB413 under the 35S promoter. All the constructs were subsequently transformed into *Agrobacterium* strain GV3101 using the freeze thaw method (Weigel and Glazebrook 2008). Primers and sequences used in this study are listed in supplementary information (Table S1 and S2).

### Plant materials

*N. benthamiana* plants were grown in a potting mix and maintained in a growth chamber at 23 °C with a 16-h day and 8-h light cycle and 70% humidity. One-month-old plants were subjected to leaf-spot infiltration with *Agrobacterium* cells harboring different constructs to test the effect of the VSRs HcPro and P19 on Cas13-dependent and Cas13-independent interference against TuMV-GFP and PDS (Fig. S3 and S4).

### Agroinfiltration of *N. benthamiana*

Binary constructs harboring LbuCas13 with either TuMV targeting guides, PDS targeting guides or non-targeting guides (m-guides) or HcPro, P19, GUS or TuMV-GFP infectious clone (Garcia et al. 2010) were individually transformed into the chemically competent *Agrobacterium tumefaciens* strain GV3101. Single colonies of each construct were inoculated into LB media supplemented with antibiotics and grown overnight at 28 °C. The next day, the cultures were centrifuged and suspended in agroinfiltration buffer (10 mM MgCl2, 10 mM MES buffer (pH 5.7) supplemented with 100 µM acetosyringone. The cultures were incubated at ambient temperature for 2–3 h. For the TuMV assay, *Agrobacterium* cells harboring LbuCas13 with TuMV targeting m-guide (OD_600_ 1.0) were mixed with either HcPro (OD_600_ 0.5), P19 (OD_600_ 0.5) or GUS (OD_600_ 0.5) and infiltrated into the adaxial side of four-week-old *N. benthamiana* leaves with the help of a 1.0 ml needleless syringe. Two days later, *Agrobacterium* cells harboring TuMV-GFP were infiltrated into the same areas at an OD600 of 0.3. Similarly, for the PDS assay, *Agrobacterium* cells harboring Cas13 with PDS-targeting m-guide (OD_600_ 1.0) were mixed with either HcPro (OD_600_ 0.5), P19 (OD_600_ 0.5) or GUS (OD_600_ 0.5) and infiltrated into the adaxial side of four-week-old *N. benthamiana* leaves. Agroinfiltrated leaf spots expressing TuMV-GFP were illuminated with a hand-held UV lamp (Fisher Scientific, Waltham, MA) and imaged using a Nikon camera.

### RNA isolation, cDNA synthesis, qRT‒PCR and northern blotting

Total RNA was isolated from agroinfiltrated leaf samples using Trizol (Ambion, Carlsbad, CA) according to the method described in Sharma et al. 2015. For first-strand cDNA synthesis, DNase-treated 1.0 µg of total RNA was reverse transcribed using random hexamers or oligo(dT20) and SuperScript II reverse transcriptase (Thermo Fisher Scientific, Waltham, MA) according to the manufacturer’s instructions. RT‒qPCR was performed using SYBR Select Master Mix (Applied Biosystem) and gene-specific primers (Supplementary File 1) for PDS. The EF1α gene was used as an internal housekeeping gene (Liu et al. 2012). The experiments were repeated twice with three biological, and two technical replicates and the relative expression values were plotted using R. Northern blotting was performed according to the procedure described in Sharma et al. 2022. For detection of the PDS transcript, 15 μg of total RNA was separated on a denaturing 1.0% agarose gel and blotted on a Hybond-N + membrane (Roche, Basel, Switzerland) using the capillary method. Later, the membrane was exposed to UV light for RNA crosslinking. A DIG-labeled PDS-specific probe was synthesized using a PCR DIG probe synthesis kit (Sigma, St. Louis, MO) according to the kit’s protocol. The membrane was hybridized with the probe in a hybridization buffer at 42℃ overnight, followed by stringent washes at 55℃.The membrane was treated with anti-DIG-AP conjugate antibody (Millipore Sigma, Burlington, MA) and washed to remove excess antibody. The membrane was treated with CSPD star solution (Millipore Sigma, Burlington, MA), and the signals were detected using a LICOR Odyssey imaging system (LI-COR Bioscience, Lincoln, NE) (Fig. S5, raw data).

### Western blotting and quantification of the bands

For western blotting, agroinfiltrated leaf tissues (50 mg) were ground to a fine powder using liquid nitrogen and resuspended in protein extraction buffer (50 mM Tris–Cl, 1% β-mercaptoethanol and protease inhibitor cocktail). The samples were centrifuged, and the supernatants were transferred to fresh tubes. Total proteins were mixed with loading buffer (100 mM Tris–Cl, 20% glycerol, 4% SDS, 10% β-mercaptoethanol, and 0.2 mg/ml bromophenol blue), denatured at 95 °C for 5 min, and separated on a 12% SDS‒PAGE gel. The proteins were separated via SDS‒PAGE and transferred to PVDF membranes (GE Healthcare, Chicago, IL) using a semidry blotting apparatus (Bio-Rad, Hercules, CA). Membrane blocking and antibody incubation were performed using an iBind western device (Thermo Fisher Scientific, Waltham, MA) according to the manufacturer’s instructions. Finally, the membrane was treated with ECL Select Western blotting detection reagent (GE Healthcare, Chicago, IL) and scanned with a LICOR Odyssey imaging system (LI-COR Bioscience, Lincoln, NE). The quantification of the western blot bands was performed using ImageJ software (https://imagej.nih.gov/ij/) (Fig. S6, raw data).

### Statistical analysis

Statistical comparisons were made using one-way ANOVA in R, and multiple comparisons of treatments by means of Tukey were performed using the HSD.test function from the Agricolae R package. Treatments with the same letter are not significantly different.

## Supplementary Information

Supplementary Material 1.

Supplementary Material 2.

## Data Availability

All data generated or analyzed in this research are included in the main article and the supplementary information document.

## Abbreviations

cRNA: CRISPR RNA
HcPro: Helper component proteinase
TuMV: Turnip mosaic virus
TBSV: Tomato bushy stunt virus
PDS: Phytoene desaturase
VSR: Viral suppressors of RNAi
GIGS: Guide-induced gene silencing
