# Supplementary material for: Plant viral suppressors HcPro and P19 inhibit CRISPR-Cas13-mediated transcript interference
Source: Stress Biol. 2026 Sep 28;6(1):69. doi: 10.1007/s44154-026-00339-9 (PMC13620101; doi:10.1007/s44154-026-00339-9)
Supplement: Supplementary file 1 [file 44154_2026_339_MOESM1_ESM.pdf]

Supplementary Information

Plant viral suppressors of RNA silencing inhibit CRISPR-Cas13a-mediated transcript interference

Sharma VK[1], Marla S[2] and Cook DE[3]*

1. Department of Forest Ecosystem and Society, Oregon State University, Corvallis, OR, USA

2. Department of Agronomy, Kansas State University, Manhattan, KS, USA

3. Department of Plant Pathology, Kansas State University, Manhattan, KS, USA.

*Correspondence (decook@ksu.edu)

Supplemental Figures

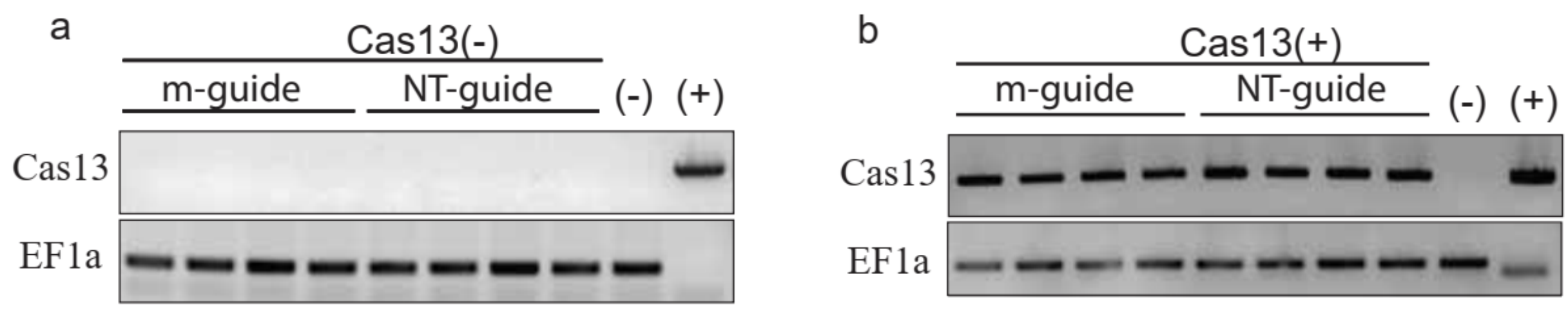

Figure S1. **Detection of LbuCas13 expression. RT-PCR detection of** the Cas13 transcript in Cas13-independent (a) and Cas13-mediated (b)TuMV-targeting assays (b). The endogenous transcript for elongation factor 1 alpha (EF1α) is shown as a control.

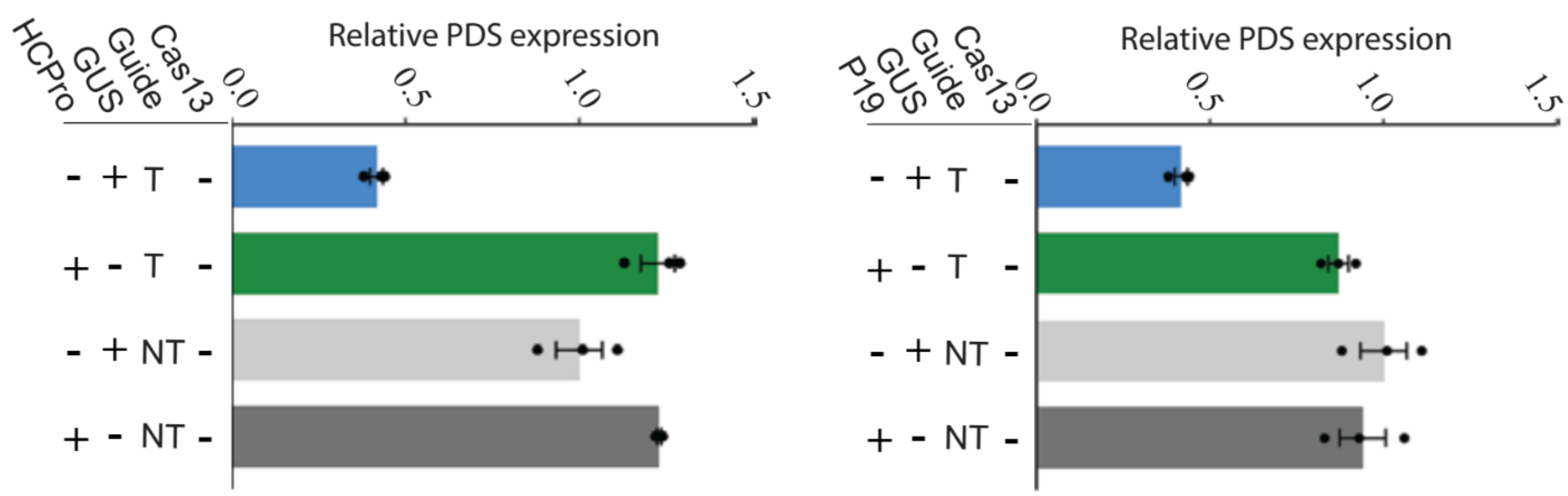

Figure S2. **The VSRs HcPro and P19 block Cas13-independent endogenous transcript interference**. Quantification of the PDS transcript using qRT–PCR. Three independent leaves were used for PDS quantification, and each data point is shown as a black dot with the standard deviation on a bar plot. The samples were standardized to the EF1α endogenous transcript and normalized to the samples expressing GUS and NT-guide. The left plot shows the expression of HcPro, and the right plot shows the expression of P19.

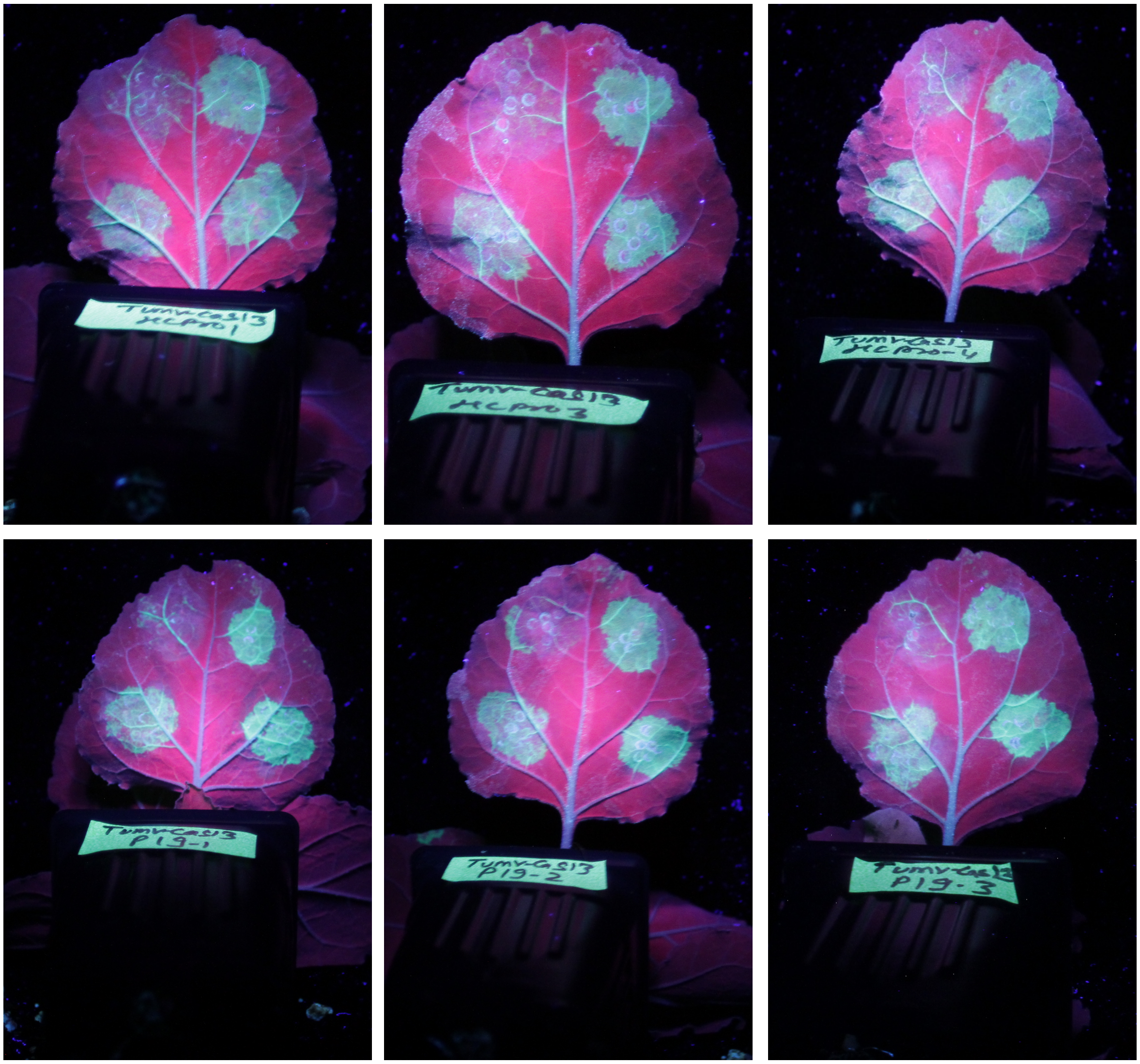

Fig S3. **Uncropped images of *N. benthamiana* plants in Cas13-mediated TuMV assays**. Top panel shows three different plants for Cas13-mediated TuMV-GFP targeting assays with HcPro, and the bottom panel shows three different Plants for Cas13-independent assays with P19. Experimental details are same as in the figure 2b and 3b.

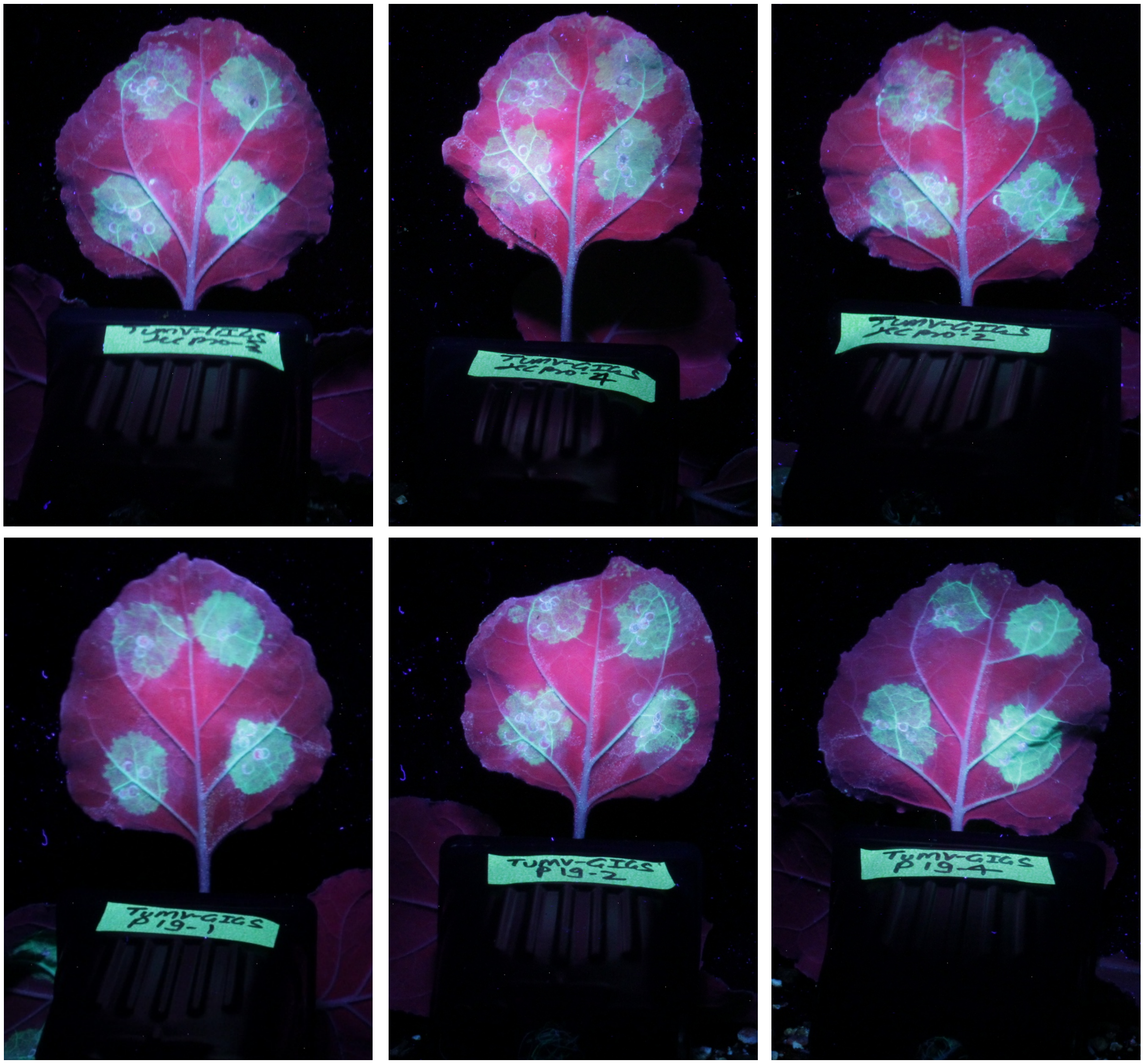

Fig S4. **Uncropped images of *N. benthamiana* plants in Cas13-independent TuMV assays**. Top panel shows three different plants for Cas13-independent TuMV-GFP targeting assays with HcPro, and the bottom panel shows three different Plants for Cas13-independent assays with P19. Experimental details are same as in the figure 2b and 3b.

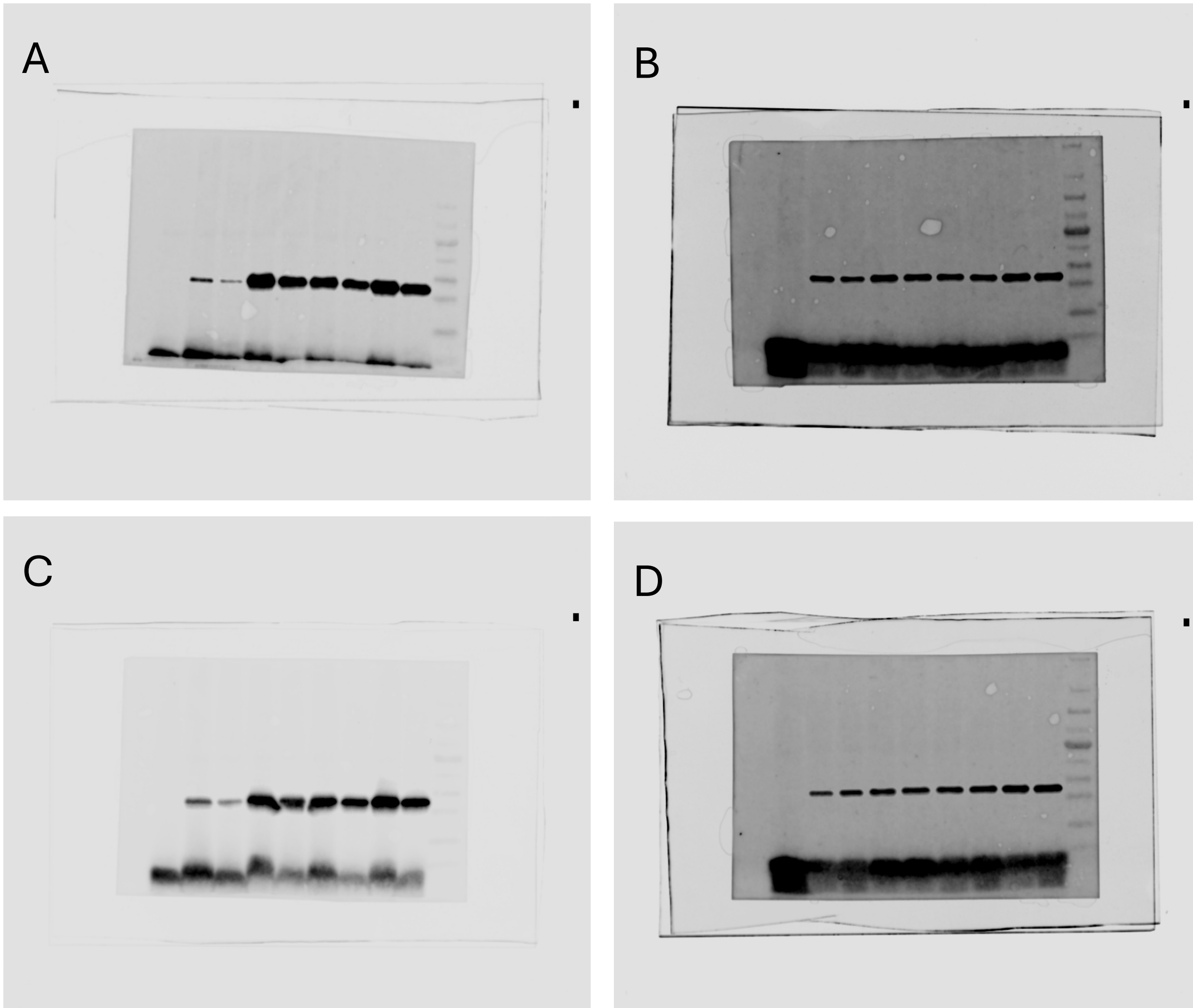

Fig. S5. Uncropped western blots. A and B blots show GFP detection in Cas13-mediated and Cas13-independent assays with HcPro respectively and B and D blots show GFP detection Cas13-mediated and Cas13-independent assays with P19.

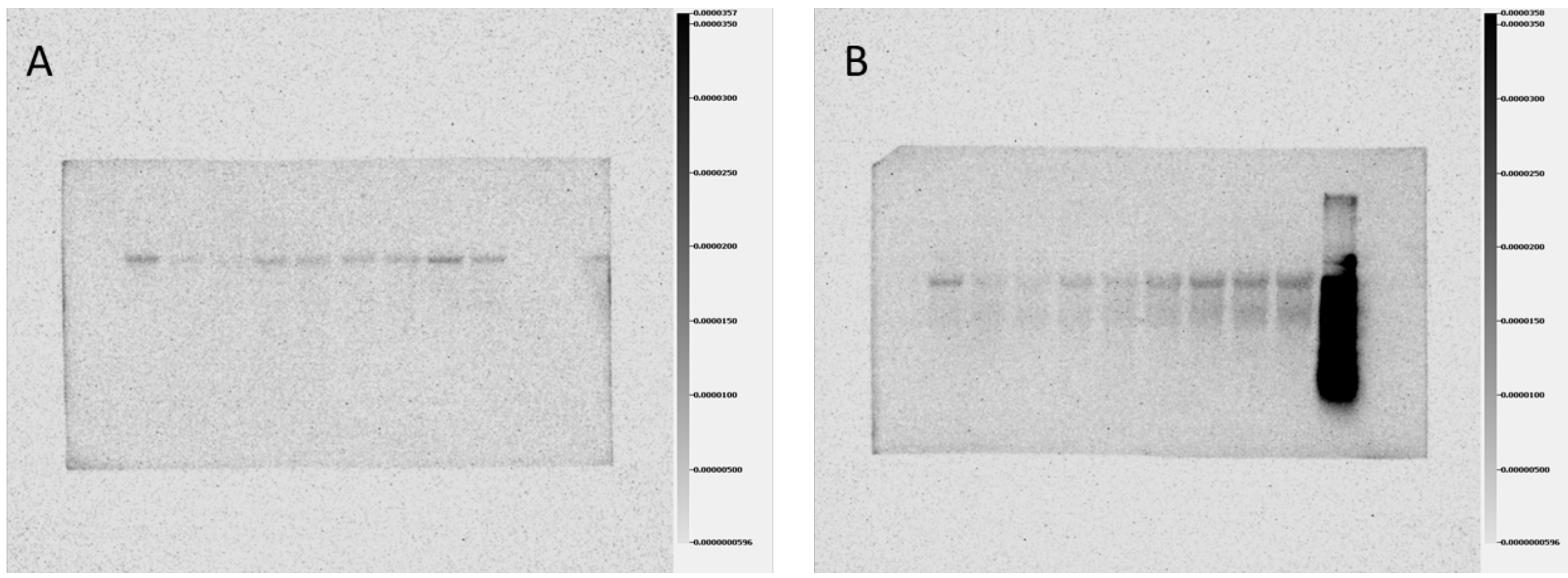

Fig S6. Uncropped northern blots. A shows PDS transcript detection in Cas13-mediated assays with HcPro and B shows PDS detection in Cas13-mediated PDS targeting assays with P19. The first lane in each blot represents RNA sample isolated from non-infiltrated leaf which is not shown in the figure 4 c, d.
