# Supplementary material for: Plant viral suppressors HcPro and P19 inhibit CRISPR-Cas13-mediated transcript interference
Source: Stress Biol. 2026 Sep 28;6(1):69. doi: 10.1007/s44154-026-00339-9 (PMC13620101; doi:10.1007/s44154-026-00339-9)
Supplement: Supplementary file 2 [file 44154_2026_339_MOESM2_ESM.pdf]

Supplemental Tables

Table S1: Sequences of TuMV, PDS and non-targeting multiguides

Name of m-guide	Sequence of m-guide	Purpose
PDS-m-guide	AACAGTCCAATTTGGGGCATTTTATTGAACAAGATTTAGACCACCCCAAAAATGAAGGGGACTAAAACATCGAAAGCTCGTCAGGGTTTATGAAGTTGATTTAGACCACCCCAAAAATGAAGGGGACTAAAACAGAAGTAACTCGTAATCCTGTACAATAGCTTTTTTT	For Nb PDS targeting
TuMV-m-guide	AACAGTTTCTGCCTTTGCCTCTTACCTTTCGCGATTTAGACCACCCCAAAAATGAAGGGGACTAAAACACTGGGAAATCTTGTTGCGAAAGGACTTCGATTTAGACCACCCCAAAAATGAAGGGGACTAAAACATTGTGTTTGCTTTAATCGTTTTGTGTATTTTTTT	For TuMV targeting
NT-m-guide	AACAGCGGTTCCATCTTCCAGCGGATAGAATGGATTTAGACCACCCCAAAAATGAAGGGGACTAAAACATCCTGAAGGCTCCTCAGAAACAGCTCTTGATTTAGACCACCCCAAAAATGAAGGGGACTAAAACACCTTTCGGTACTTCGTCCACAAACACAATTTTTTT	Non-targeting

Table S2: Oligo sequences used in this study

Oligo name	Sequence (5′-3′)	Purpose
MluI-HcP-F	GTAGGTACGCGTATGGCAGGTGCAGCGGGAGCCAA	For HcPro cloning
AvrII-HcP-R	GATGTCCCTAGGTCATCCAACGCGGTAGTGTTTC	For HcPro cloning
MluI-P19-F	TCAGTGACGCGTATGGAACGAGCTATACAAGGAAAC	For P19 cloning
AvrII-P19-R	GTCACTCCTAGGTTACTCGCTTTCTTTTTCGAAG	For P19 cloning
AatII-GUS-F	CTGCATGACGTCATGTTACGTCCTGTAGAAACCCCAAC	For GUS cloning
AvrII-GUS-R	CTGCATCCTAGGTCATTGTTTGCCTCCCTGCTGCGG	For GUS cloning
PDS3for	GGACTGGATGAGAAAGCAAGGTGT	For qRT-PCR of PDS
PDS3rev	ATCGAAAGCTCGTCAGGGTTTATG	For qRT-PCR of PDS
EF1a-FP	AGCTTTACCTCCCAAGTCATC	For qRT-PCR of EF1a
EF1a-RP	AGAACGCCTGTCAATCTTGG	For qRT-PCR of EF1a
PDS-Probe-FP	GGTCACAAACCGATATTGCTGGAGG	For PDS northern
PDS-Probe-RP	ACAGACATGTCAGCGTACACACTGAGCA	For PDS northern
Cas13-RT-For	GCGAGGGTCGATTAGTGAAAT	For Cas13 RT-PCR
Cas13-RT-Rev	CCAGGATGTCCGTTTCTGAATA	For Cas13 RT-PCR
HCP-SDM-FP	TGCACCAAATGGGTCACGGAAAC	For HcPro mutagenesis
HCP-SDM-RP	GCTGCTGTCTCGTATTGGGTGTAG	For HcPro mutagenesis
mHCP-RT-F	GCTACACCCAATACGAGACAGC	For mutant HcPro RT-PCR
mHCP-RT-R	CATCTAACAGAGTGGGCCACTTG	For mutant HcPro RT-PCR
wHCP-RT-F	GCTACACCCAATACGAGACAAGA	For wild type HcPro RT-PCR
wHCP-RT-F	CATCTAACAGAGTGGGCCACTTG	For wild type HcPro RT-PCR
